# OxInflammation at High Altitudes: A Proof of Concept from the Himalayas

**DOI:** 10.3390/antiox11020368

**Published:** 2022-02-11

**Authors:** Simona Mrakic-Sposta, Denise Biagini, Danilo Bondi, Tiziana Pietrangelo, Alessandra Vezzoli, Tommaso Lomonaco, Fabio Di Francesco, Vittore Verratti

**Affiliations:** 1 Institute of Clinical Physiology, National Research Council (IFC-CNR), 20162 Milan, Italy; simona.mrakicsposta@cnr.it (S.M.-S.); alessandra.vezzoli@cnr.it (A.V.); 2 Department of Chemistry and Industrial Chemistry, University of Pisa, 56126 Pisa, Italy; tommaso.lomonaco@unipi.it (T.L.); fabio.difrancesco@unipi.it (F.D.F.); 3 Department of Neuroscience, Imaging and Clinical Sciences, University “G. d’Annunzio” of Chieti, 66100 Chieti, Italy; danilo.bondi@unich.it (D.B.); tiziana.pietrangelo@unich.it (T.P.); 4 Department of Psychological, Health and Territorial Sciences, University “G. d’Annunzio” of Chieti, 66100 Chieti, Italy; vittore.verratti@unich.it

**Keywords:** hypobaric hypoxia, redox, inflammatory response, trekking, oxylipins, biomarkers

## Abstract

High-altitude locations are fascinating for investigating biological and physiological responses in humans. In this work, we studied the high-altitude response in the plasma and urine of six healthy adult trekkers, who participated in a trek in Nepal that covered 300 km in 19 days along a route in the Kanchenjunga Mountain and up to a maximum altitude of 5140 m. Post-trek results showed an unbalance in redox status, with an upregulation of ROS (+19%), NOx (+28%), neopterin (+50%), and pro-inflammatory prostanoids, such as PGE_2_ (+120%) and 15-deoxy-delta12,14-PGJ_2_ (+233%). The isoprostane 15-F_2t_-IsoP was associated with low levels of TAC (−18%), amino-thiols, omega-3 PUFAs, and anti-inflammatory CYP450 EPA-derived mediators, such as DiHETEs. The deterioration of antioxidant systems paves the way to the overload of redox and inflammative markers, as triggered by the combined physical and hypoxic stressors. Our data underline the link between oxidative stress and inflammation, which is related to the concept of OxInflammation into the altitude hypoxia fashion.

## 1. Introduction

High altitude exposure triggers marked physiological responses and adaptations [1]. For this reason, challenges to human homeostasis by such environmental stressors [2] provide an intriguing ecological model to reproduce physiological and pathophysiological conditions that share hypoxemia as the common denominator. In fact, altitude travel has increased massively in the last few decades, and the combination of physical activity and altitude hypoxia, as in high-altitude treks, enables medical and physiological responses to be investigated in the field [3,4].

Hypoxia-induced inflammation impacts on the immune function and causes chronic disease and high-altitude illnesses [5]. In the same vein, redox homeostasis has been widely studied as disrupted by hypoxia conditioning [6]. Hypobaric hypoxia negatively affects redox homeostasis, leading to the generation of reactive species and consequently to damage of cellular compartments (i.e., lipids and proteins) and the degeneration of antioxidant systems [7,8]. On the other hand, ascent to high altitudes can lead to an excessive inflammatory response [7,9,10] with increased levels of proinflammatory cytokines [7,9,10]. The reciprocal interplay between oxidative stress and inflammation has been termed as OxInflammation, and it is a source of biomarkers for monitoring the pre-pathological conditions triggered by environmental stressors [11].

Oxylipins are well-known markers of oxidative damage and inflammation [12] as well as powerful lipid mediators generated from both omega-3 and omega-6 polyunsaturated fatty acids (PUFAs) [13]. PUFAs act as precursors of pro-inflammatory, anti-inflammatory, and specialized pro-resolving lipid mediators (SPMs) through enzymatic oxidation reactions [14].

Pro-inflammatory mediators released at the very beginning of inflammation foster the appearance of its classic signs, whereas the switch to lipoxygenase-derived anti-inflammatory and pro-resolving mediators leads to a natural resolution of inflammation [15]. PUFAs can also undergo free radical-mediated oxidation, thus generating well-established markers of oxidative stress such as isoprostanes (e.g., 15-F_2t_-IsoP, 15-E_2t_-IsoP) [16]. High levels of F_2_-isoprostane have been observed after both acute and chronic high-altitude exposure and were correlated with increased plasma levels of total glutathione [17]. High-altitude research expeditions up to 4000–5000 m have induced a temporary increase in F_2_-isoprostane concentration compared with that observed at sea levels [18,19], thus suggesting the combined effect of altitude exposure and exercise on cellular (even nervous system-related) oxidative damage [20].

It is thus clear that high-altitude treks increase oxidative stress and trigger a pronounced inflammatory response. However, these phenomena and their interaction are currently still being characterized in depth. Our field study on high-altitude trekkers in the Himalayas explores the new concept of OxInflammation. We propose the comprehensive monitoring of the redox status and antioxidant capacity together with the measurement of a wide panel of oxylipins for a full characterization of oxidative damage and immune-inflammatory response.

## 2. Materials and Methods

This work is part of the “Kanchenjunga Exploration & Physiology” research project, which is a subset of the project “environmentally-modulated metabolic adaptation to hypoxia in altitude natives and sea-level dwellers: from integrative to molecular (proteomics, epigenetics, and ROS) level”. The project was approved by the Ethical Review Board of the Nepal Health Research Council (NHRC, ref. no. 458). All the study procedures complied with the ethical standards of the Helsinki declaration, and written informed consent was obtained before sample and data collection.

The group of trekkers was composed by one female and five males, aged 40 ± 20 years, with a BMI of 26 ± 3 kg/m^2^. Participants completed a trek of 300 km in 19 days along a demanding route in the Kanchenjunga Mountain (Himalayas, Nepal), up to a maximum altitude of 5140 m (North Base Camp).

Blood was drawn from the antecubital vein two weeks before the start of the trip and the day after the end of the Himalayan trek. The first blood sample was collected in Vacutainer^®^ tubes and immediately centrifuged (3000 rpm × 10 min). The second sample was stored at −5 °C during transport to Italy for later analyses. For the oxylipin analyses, the antioxidant butylhydroxytoluene (BHT, 15 mg/mL in methanol) was added (1:100, BHT:sample) before storage to preserve PUFAs from in vitro lipid peroxidation [21]. First-void urinary samples were immediately frozen at −20 °C and stored until analysis. Daily food, water, MUFA, and PUFA intake were self-recorded by the subjects, on three non-consecutive days during the trek [22].

An X band electron paramagnetic resonance spectroscopy (9.3 GHz) (E-Scan-Bruker BioSpin, GmbH, Billerica, MA, USA) was used to assess total ROS production and total antioxidant capacity (TAC) at pre- and post-high-altitude trek. 

CMH (1-hydroxy-3-methoxycarbonyl-2,2,5,5-tetramethylpyrrolidine) spin probe was used for ROS determination. 50 μL of sample were treated with CMH solution (1:1). 50 μL of the obtained solution were put in a glass EPR capillary tube (Noxygen Science Transfer & Diagnostics, Elzach, Germany) that was placed inside the cavity of the E-scan spectrometer for data acquisition with the following parameters of acquisition: microwave frequency 9.652 GHz; modulation frequency 86 kHz; modulation amplitude 2.28 G; sweep width 60 G, microwave power 21.90 mW, number of scans 10; and receiver gain 3.17⋅101. A stable radical CP · (3-carboxy2,2,5,5-tetramethyl-1-pyrrolidinyloxy) was used as an external reference to convert ROS determinations in absolute quantitative values (μmol•min^−1^), as previously indicated [7,8,23].

TAC was measured using 1,1-diphenyl-2-picrylhydrazyl (DPPH•), a free radical compound soluble and stable in ethanol. 5 μL of plasma were added to 45 μL of buffer solution (5 mM potassium phosphate, pH 7.4 containing 0.9% sodium chloride) then reaction was initiated by the addition of 50 μL of DPPH• as a source of free radicals, as previously indicated [24,25,26]. Reaction mixture was incubated for 30 min at dark room temperature (for the photochemical effect on DPPH) and then 50 μL of the obtained solution was put in the glass EPR capillary tube. The calculated antioxidant capacity was expressed in terms of Trolox equivalent antioxidant capacity (TAC, mM). 

All samples were stabilized at 37 °C with “Bio III” unit (Bio III—Noxigen Science Transfer & Diagnostics GmbH, Germany), interfaced to the spectrometer. Spectra were recorded and analyzed using Win EPR software (2.11 version) standardly supplied by Bruker.

Assessment methods have been previously described [23,26,27]. Total aminothiols (Cys: cysteine; CysGly: cysteinylglycine; Hcy: homocysteine; and GSH: glutathione) were measured by high-performance liquid chromatography (HPLC) according to previously validated methods [28,29]. NO metabolites (NOx) concentrations were determined in urine via a colorimetric method based on the Griess reaction, using a commercial kit (Cayman Chemical, Ann Arbor, MI, USA) as previously described [26,30]. Urinary creatinine, neopterin, and uric acid concentrations were measured by the HPLC method as previously described [7].

The MS-based targeted profiling of 25 plasma oxylipins—i.e., isoprostanes (15-F_2t_-IsoP and 15-E_2t_-IsoP), prostanoids (TXB_2_, PGE_2_ and 15-deoxy-delta12,14-PGJ_2_), hydroxy- and epoxy-fatty acids (8,9-EET, 11,12-EET, 14,15-EET, 13-HODE, 5-HETE, 12-HETE, 15-HETE, 20-HETE, 8,9-DiHETE, 11,12-DiHETE, 14,15-DiHETE), and of omega-3/omega-6 PUFAs (AdA, EPA, alpha-LA, la, DHA, DPA, AA) was performed using micro-extraction by packed sorbent (MEPS) liquid chromatography tandem mass spectrometry (MEPS-LC-MS/MS) platform [31,32]. Briefly, plasma proteins were precipitated by the sequential addition of salts (i.e., 250 µL of CuSO_4_·5 H_2_O 10% *w*/*v* and 250 µL of Na_2_WO_4_·2 H_2_O 12% *w*/*v*) and acetonitrile (500 µL) to the plasma sample (500 µL). The supernatant was then diluted (1:6 *v*/*v*) with water and subjected to MEPS extraction. The methanolic extract was directly injected into the UHPLC-MS/MS system and oxylipins were analyzed as described elsewhere [33].

Statistical analyses were carried out with the open-source R-based software Jamovi v. 1.6.18.0. Data were initially checked for normality with the Shapiro–Wilk test, then LEU-B_4_, 8,9-DiHETE, 8,9-EET, DPA and NOx data were log-transformed, whereas uric acid data were loglog-transformed; PGD_2_ was removed from the analysis due to missing data. After a Kolmogorov–Smirnov check for normality, a series of paired sample *t*-tests were carried out. The effect size (Cohen’s d) was adjusted to the unbiased value for low sample size as dunb = d[1-3/(4df-1)], then graphs were created using Prism v. 9.2.0 (GraphPad Software, San Diego, CA, USA).

Data were also analyzed by a multivariate exploratory method (principal component analysis, PCA [34]) using MetaboAnalyst v. 5.0 (Wishart Research group, The Metabolomics Innovation Centre (TMIC), University of Alberta, Canada) [35,36]. The dataset included the plasma levels of 23 oxylipins and the 11 markers of both the redox status and the antioxidant capacity. Two out of 25 oxylipins (i.e., 15-E_2t_-IsoP and PGD_2_) were excluded from the dataset as the concentrations were below the limit of quantification for more than 50% of samples. The original data were pre-processed (data integrity and missing value check) and normalized (i.e., square root transformation and data autoscaling) prior to the multivariate analysis [37].

## 3. Results

The average daily intake of participants, estimated from food diaries loaded into an ad-hoc web database [22], included 39 ± 9 g (13 ± 2% of the total energy intake) of PUFAs among other nutrients and 3000 ± 500 g of water. The trekkers suffered the combined stress of physical exercise and hypoxia, as elsewhere reported with a suppression of the hypothalamus-pituitary gonadal axis and altered thyroid metabolic function [38], a weight loss and a reduction in total body water [22], while the mood disturbance scores were lower at high altitude [39].

**Figure 1:**
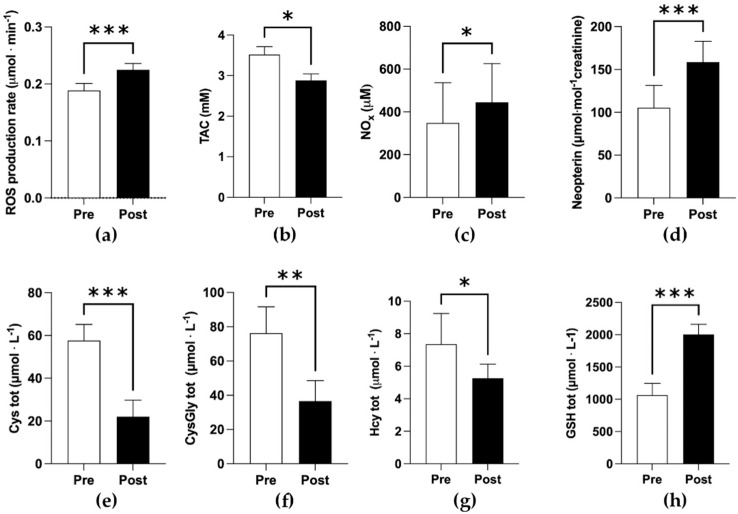
Bar plots (mean ± SD) of redox results. (**a**) ROS production rate; (**b**) TAC; (**c**) NOx; (**d**) neopterin; (**e**–**h**) total concentration of aminothiols (*Cys*, cysteine; *CysGly*, cysteinylglycine; *Hcty*, homocysteine; *GSH*, glutathione) collected pre- and post- high-altitude trek (* *p* < 0.05, ** *p* < 0.01, *** *p* < 0.001, significantly different).

Significant increases post-trek of ROS production rate (0.18 ± 0.01 vs. 0.22 ± 0.01 μmol min^−1^) (Figure 1a), NOx (300 ± 200 vs. 400 ± 200 μM) (Figure 1c), total Hcy and GSH (5.3 ± 0.9 vs. 7 ± 2; 1100.0 ± 200 vs. 2000 ± 200 μmol L^−1^ respectively) were detected (Figure 1g,h). Conversely, TAC (3.5 ± 0.2 vs. 2.9 ± 0.2 mM), and total Cys and CysGly (58 ± 8 vs. 22 ± 8; 80 ± 10 vs. 40 ± 10 μmol L^−1^ respectively) significantly decreased at post. Changes in uric acid levels were far from significance (0.4 ± 0.4 vs. 0.4 ± 0.5 μmol L^−1^, respectively). While at post, a significant increase in neopterin was observed (100 ± 30 vs. 160 ± 20 μmol mol^−1^ creatinine, Figure 1d). The overall statistical results are reported in Table 1.

**Figure 2:**
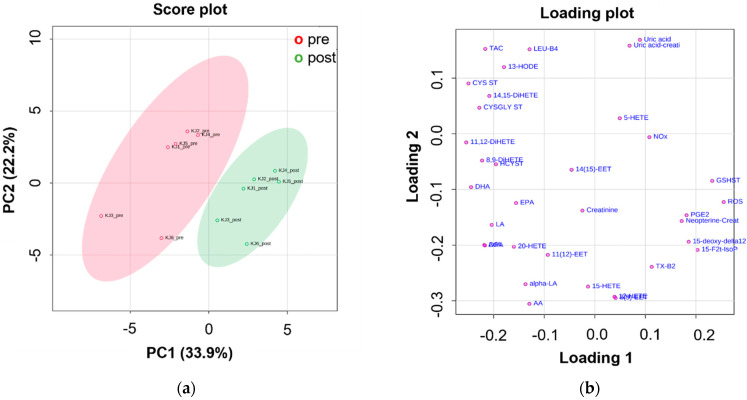
(**a**) Score plot and (**b**) loading plot of PCA performed on the overall dataset. Red and green symbols represent trekkers pre- and post-high-altitude trek, respectively. Data were pre-processed (i.e., square root transformation and data autoscaling) prior to the multivariate analysis.

The PCA also highlighted a clear separation among samples collected pre- and post- high-altitude trek. Figure 2 shows the score (a) and loading plots (b) of PCA for the pre-processed data. The two lowest-order principal components accounted for a total explained variance of about 56%. PC1 scores provided the main contribution to the separation between the two sample classes, with pre-trek samples (red circle) showing negative score values, whereas the post-trek (green circle) showed positive scores (Figure 2a). Post-trek samples were mostly characterized by an upregulation of ROS, NOx, total GSH, urinary neopterin, pro-inflammatory prostanoids (e.g., PGE_2_ and 15-deoxy-delta12,14-PGJ_2_), and isoprostane 15-F_2t_-IsoP (Figure 2b). These samples were associated with low levels of TAC, total Gly and CysGly, omega-3 PUFAs (e.g., DHA, EPA, DPA) and, to a lesser extent, anti-inflammatory CYP450 EPA-derived mediators such as DiHETEs (Figure 2b). Oxylipin mean concentration levels pre- and post-trek are reported in Table 2.

## 4. Discussion

The combined effect of high altitude and physical exercise on oxidative stress has long remained unknown [40], and the limited knowledge available derives from a small number of recent field and lab simulation studies [41]. Omics sciences are however now helping to shed new light on the complex metabolic rearrangement caused by altitude hypoxia [42,43]. Muscles working under hypoxic environments are overstressed, and the deterioration of antioxidant systems leads to the loss of protective molecular processes [44]. Indeed, altered redox homeostasis as compromised by hypobaric hypoxia determines an impairment of muscle performance and paves the way to skeletal muscle atrophy [45].

Here, we combined a high-throughput analytical platform for the targeted metabolomic profiling of oxylipins to the measurement of ROS and antioxidants for a comprehensive characterization of high-altitude induced redox homeostasis disruption and inflammatory response. In line with the literature [7,8,10,46], we found that high altitude increases the production of ROS and decreases the antioxidant capacity, thus modifying the weighing pan, as suggested by the increase in ROS, NOx, oxidative damage biomarkers (i.e., 15-F_2t_-IsoP), and unbalanced redox status (i.e., total Cys and CysGly). We also observed a pronounced immune-inflammatory response at the post-trek sampling point, with a marked increase in urinary neopterin (a well-known marker of cellular immune system activation), the simultaneous upregulation of pro-inflammatory prostanoid levels (e.g., TXB_2_, PGE_2_, and 15-deoxy-delta12,14-PGJ_2_) and downregulation of omega-3 PUFAs and anti-inflammatory DiHETEs. These results clearly suggest an acute OxInflammation outbreak in response to the hypoxic trek, where muscle damage may have led to increased circulating PUFAs and ROS-mediated peroxidation [47].

The overproduction of ROS represents a well-established response to exercise [48,49,50]. High-altitude exercise induces oxidative stress and muscle fatigue to a greater extent since hypoxia increases ROS production and decreases antioxidant capacity [51,52,53]. Humans show complex multiple protective systems to maintain the redox homeostasis, starting with transitory effects that are highly regulated and modulated within minutes to longer lasting ones (over periods of days to years) [54]. Since redox signaling is required for numerous physiological processes, a disruption in redox homeostasis can regulate detrimental signaling pathways and produce harmful effects on several substrates [55].

Among lipids, PUFAs are majorly affected by free radical peroxidation, thus generating isoprostanes as principal end-products. Isoprostanes, such as 15-F_2t_-IsoP and 15-E_2t_-IsoP, have been found to amplify the vasoconstriction induced by angiotensin II (Ang-II) and endothelin-1 (ET-1) in hypoxic conditions [56]. The latter are strictly related to vascular xanthine oxidoreductase activity, which underlies hypoxia-induced oxidative damage combined with a reduction in mitochondrial redox potential [57], an increase in catecholamine production [58], and the activation of phospholipase A2.

ET-1 also induces inflammatory processes in the vascular wall and to promote the expression of proinflammatory cytokines [59], which in turn activates the production of prostaglandins through cyclooxygenases (COX) enzymes. Prostaglandins are pro-inflammatory and vasodilator immuno-mediators originating from arachidonic acid, whose plasma concentrations increase in response to high altitudes [60]. The release of COX-metabolites seems to be a key factor contributing to mountain sickness symptoms [61]. More broadly, hypoxia induces a substantial increase in the plasma concentration of most PUFA-derived eicosanoids, also produced from cytochrome P450 (CYP450) and lipoxygenase (LOX) enzymes [62]. Among LOX-metabolites, SPMs promote the resolution of inflammation thus enhancing the overall host response [63]. Nevertheless, whether the combination of high-altitude and exercise affects the resolution of inflammation or the release and activity of SPMs is still not fully clear, thus representing an interesting topic for future investigations.

## 5. Conclusions

Current physiological methods and molecular biological tools are improving and extending altitude physiology, allowing accurate measurements of the variables of interest and the responsible mechanisms [64]. *Omics* approaches are entering into the field of high-altitude medicine, since a comprehensive understanding of the biochemical response to hypoxia can define novel strategies for dealing with hypoxia-susceptible biomarkers in pathophysiology [65].

The wide set of potential biomarkers highlighted by this proof of concept entices for the design of case-control, hypothesis-testing studies to be proposed for enriching the theoretical and applicative insights in environmental and exercise physiology. Individual variability in hypoxia responses is growing interest for 5P medicine since oxygen deficiency is a key factor in many pathological processes. Therefore, besides the biomarkers suggested in the literature—such as hypoxia-inducible factor (HIF)-1, heat-shock protein 70 (HSP70), and nitric oxide (NO) [66]—oxylipins (as the ones targeted by this study) can be used to investigate the individual reactions to hypoxia.

## Figures and Tables

**Table 1:** Statistical results.

	Test	*p*-Value	Cohen’s d Unbiased	95% CI	
				*Lower*	*Upper*
**15-F_2t_-IsoP**	Wilcoxon	0.035	−2.041	−4.046	−0.746
**TX-B_2_**	Student	0.073	−0.777	−1.869	−0.080
**15-E_2t_-IsoP**	Wilcoxon	0.438	0.300		
**PGE_2_**	Student	0.043	−0.927	−2.110	−0.032
***14,15-DiHETE***	*Student*	*0.073*	*0.777*	*−0.081*	*1.868*
***11,12-DiHETE***	*Student*	*0.075*	*0.772*	*−0.085*	*1.860*
***LEU-B_4_***	*Student*	*0.001*	*2.192*	*0.833*	*4.339*
***8,9-DiHETE***	*Student*	*0.083*	*0.744*	*−0.106*	*1.817*
**15-deoxy-delta12,14-PGJ_2_**	Student	0.022	−1.128	−2.446	−0.174
***20-HETE***	*Student*	*0.043*	*0.929*	*0.033*	*2.113*
***13-HODE***	*Student*	*0.136*	*0.611*	*−0.213*	*1.611*
**15-HETE**	Student	0.405	0.312		
**12-HETE**	Student	0.226	0.475		
**5-HETE**	Student	0.564	0.212		
***14,15-EET***	*Student*	*0.039*	*0.953*	*0.051*	*2.153*
**11,12-EET**	Wilcoxon	0.710	0.205		
**8,9-EET**	Student	0.035	−0.986	−2.208	−0.075
***AdA***	*Student*	*0.122*	*0.639*	*−0.189*	*1.654*
***EPA***	*Student*	*0.115*	*0.656*	*−0.176*	*1.679*
**alpha-LA**	Wilcoxon	0.844	0.219		
***DHA***	*Student*	*0.008*	*1.486*	*0.410*	*3.067*
**AA**	Student	0.610	0.187		
***DPA***	*Student*	*0.061*	*0.826*	*−0.043*	*1.946*
***LA***	*Student*	*0.018*	*1.188*	*0.215*	*2.548*
**Creatinine**	Student	0.763	−0.109		
**Neopterin/Creatinine**	Student	0.010	−1.396	−2.909	−0.353
**Uric acid**	Student	0.296	−0.402		
**Uric acid/Creatinine**	Wilcoxon	0.688	0.044		
**NOx**	Student	0.020	−1.156	−2.493	−0.194
***Cys ST***	*Student*	*<0 .001*	*2.458*	*0.983*	*4.829*
***CysGly ST***	*Student*	*0.002*	*1.930*	*0.681*	*3.863*
***Hcy ST***	*Student*	*0.029*	*1.039*	*0.112*	*2.295*
**GSH ST**	Student	<0.001	−3.607	−6.967	−1.600
**ROS**	Student	< 0.001	−4.134	−7.957	−1.873
***TAC***	*Student*	*<0.001*	*2.989*	*1.273*	*5.812*

Note: Italics represent those values that decreased from pre to post expedition (as also expressed by the positive sign of the effect size); grey-background rows represent the significant results (*p* < 0.05), and among them dark-grey rows represent the most consistent findings (absolute range of 95% C.I. of effect size over 0.4).

**Table 2:** Oxylipin mean concentration levels measured pre- and post-high-altitude trek.

Compound *	Pre-Altitude Trek		Post-Altitude Trek	
	*Mean*	*SD*	*Mean*	*SD*
***15-F_2t_-IsoP***	**0.024**	**0.006**	**0.06**	**0.01**
*TX-B_2_*	0.6	0.5	1.1	0.4
***PGE_2_***	**0.05**	**0.03**	**0.11**	**0.07**
*14,15-DiHETE*	0.008	0.005	0.004	0.001
*11,12-DiHETE*	0.008	0.006	0.003	6 × 10^−4^
***LEU-B_4_***	**0.1**	**0.2**	**0.03**	**0.03**
*8,9-DiHETE*	0.008	0.004	0.006	7 × 10^−4^
***15-deoxy-Δ12,14-PGJ_2_***	**0.006**	**0.006**	**0.02**	**0.02**
***20-HETE***	**0.03**	**0.02**	**0.022**	**0.008**
*13-HODE*	20	6	13	4
*15-HETE*	0.3	0.2	0.4	0.2
*12-HETE*	20	20	30	6
*5-HETE*	0.3	0.2	0.27	0.06
***14,15-EET***	**0.015**	**0.006**	**0.012**	**0.005**
*11,12-EET*	0.004	0.001	0.003	0.002
***8,9-EET***	**0.02**	**0.02**	**0.026**	**0.009**
*AdA*	4000	3000	3000	1000
*EPA*	5000	3000	2500	900
*alpha-LA*	20,000	10,000	16,000	9000
***DHA***	**20,000**	**7000**	**14,000**	**5000**
*AA*	18,000	9000	17,000	4000
*DPA*	4000	3000	3000	1000
***LA***	**23,000**	**3000**	**20,000**	**3000**

* All concentration levels are reported as ppb. Significant changes (*p* < 0.05) are reported in bold.

## Data Availability

Data is contained within the article.
